# Study on mechanism of matrine in treatment of COVID-19 combined with liver injury by network pharmacology and molecular docking technology

**DOI:** 10.1080/10717544.2021.1879313

**Published:** 2021-02-01

**Authors:** Fangzhou Liu, Yuanbai Li, Yang Yang, Meng Li, Yu Du, Yiying Zhang, Jing Wang, Yujing Shi

**Affiliations:** a Institute of Information on Traditional Chinese Medicine, China Academy of Chinese Medical Sciences, Beijing, China; b Institute of Chinese Materia Medica, China Academy of Chinese Medical Sciences, Beijing, China

**Keywords:** Matrine, COVID-19, liver injury, network pharmacology, molecular docking, real-time RT-PCR

## Abstract

The aim of the present study was to investigate the pharmacological mechanism of matrine in treatment of COVID-19 combined with liver injury. Potential targets related to matrine, COVID-19 and liver injury were identified from several databases. We constructed PPI network and screened the core targets according to the degree value. Then, GO and KEGG enrichment were carried out. Molecular docking technology was used to verify the affinity between matrine and the crystal structure of core target protein. Finally, real-time RT-PCR was used to detect the effects of matrine on hub gene expression in liver tissue of liver injury mice and lung tissue of lung injury mice to further confirm the results of network pharmacological analysis. The results show that six core targets including AKT1, TP53, TNF, IL6, BCL2L1 and ATM were identified. The potential therapeutic mechanism of matrine on COVID-19 combined with liver injury is closely related to regulate antiviral process, improve immune system and regulate the level of inflammatory factors. Molecular docking showed that matrine could spontaneously bind to the receptor protein and had strong binding force. Real-time RT-PCR demonstrated that matrine could significantly reduce the expression of AKT1, TP53, TNF, IL6 and ATM in mice with liver injury or lung injury (P < 0.05), and increase the expression of BCL2L1 to a certain extent (P > 0.05). Our results indicate that matrine can achieve simultaneous intervention of COVID-19 combined with liver injury by multi-dimensional pharmacological mechanism.

## 1. Introduction

Novel coronavirus pneumonia caused by the novel coronavirus has been listed as an international public health emergency by the World Health Organization (Wu et al., 2020). The virus has spread around the world, causing fever, severe respiratory diseases, and pneumonia, and has posed a major threat to public health (Wang et al., 2020; Zhu et al., 2020).

It is worth noting that in addition to the respiratory system, a large number of COVID-19 patients also show various degrees of clinical features of liver injury (Du et al., 2020; Pan et al., 2020). Chen et al. found that among 99 patients with COVID-19, 43 patients had different degrees of liver function injury (Chen et al., 2020). Clinical data from the Fifth Medical Center of PLS General Hospital, Beijing, China shows that 2–11% of patients with COVID-19 had liver complications, while 14–53% of patients reported abnormal levels of alanine aminotransferase and aspartate aminotransferase [AST] during disease progression, and patients with severe COVID-19 appear to have a higher rate of abnormal liver function (Zhang et al., 2020). In a study by Huang et al., the elevation of AST was observed in eight of 13 patients [62%] in the intensive care unit [ICU] compared with seven of 28 patients [25%] who did not require care in the ICU (Huang et al., 2020). About 2–10% of COVID-19 patients have diarrhea symptoms, and SARS-CoV-2 RNA can be detected in stool and blood samples (Yeo et al., 2020). This evidence suggests that the liver may be infected with the virus. In view of this situation, ‘*The protocol for prevention, diagnosis and treatment of liver injury in coronavirus disease 2019*’ was formulated by China Digestion Association of Chinese Medical Doctor Association and Chinese Society of Hepatology of Chinese Medical Association (China Digestion Association of Chinese Medical Doctor Association, 2020). What’s more, for severe patients with COVID-19 and previous severe liver disease, especially elderly patients with comorbidities, more targeted treatment is needed. Therefore, it is of great significance to develop effective drugs for the treatment of comorbidities.

**Figure 1.**
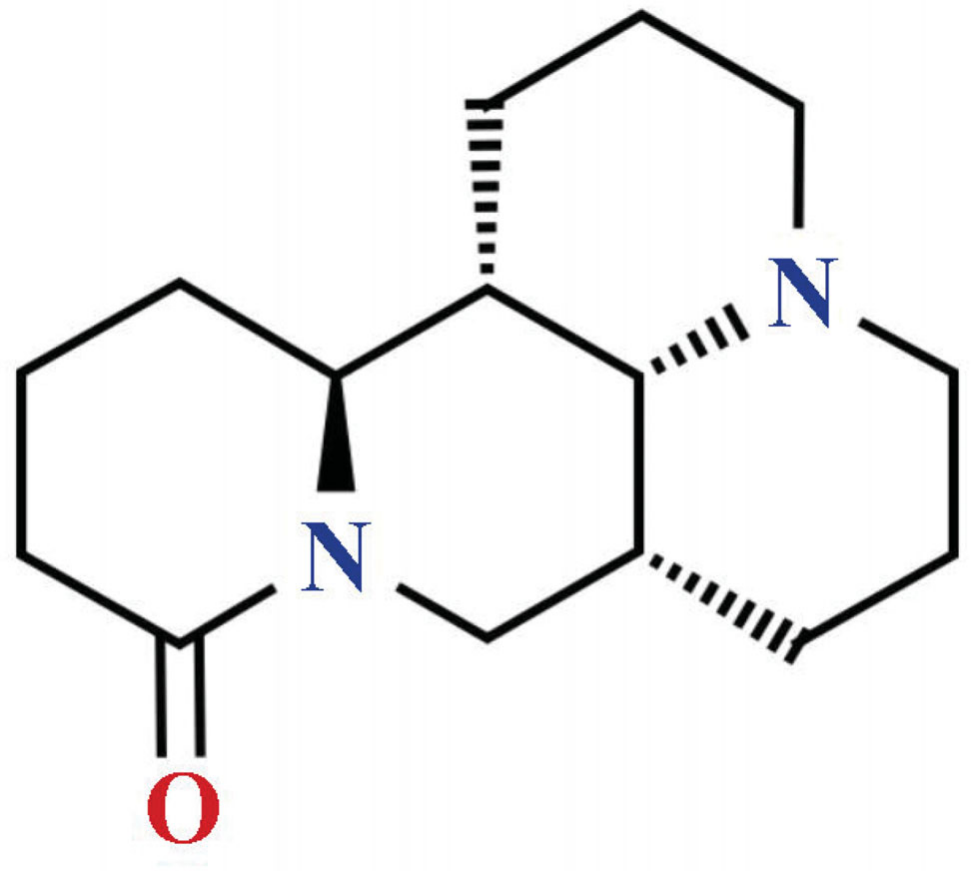
Chemical structure of matrine.

**Figure 2.**
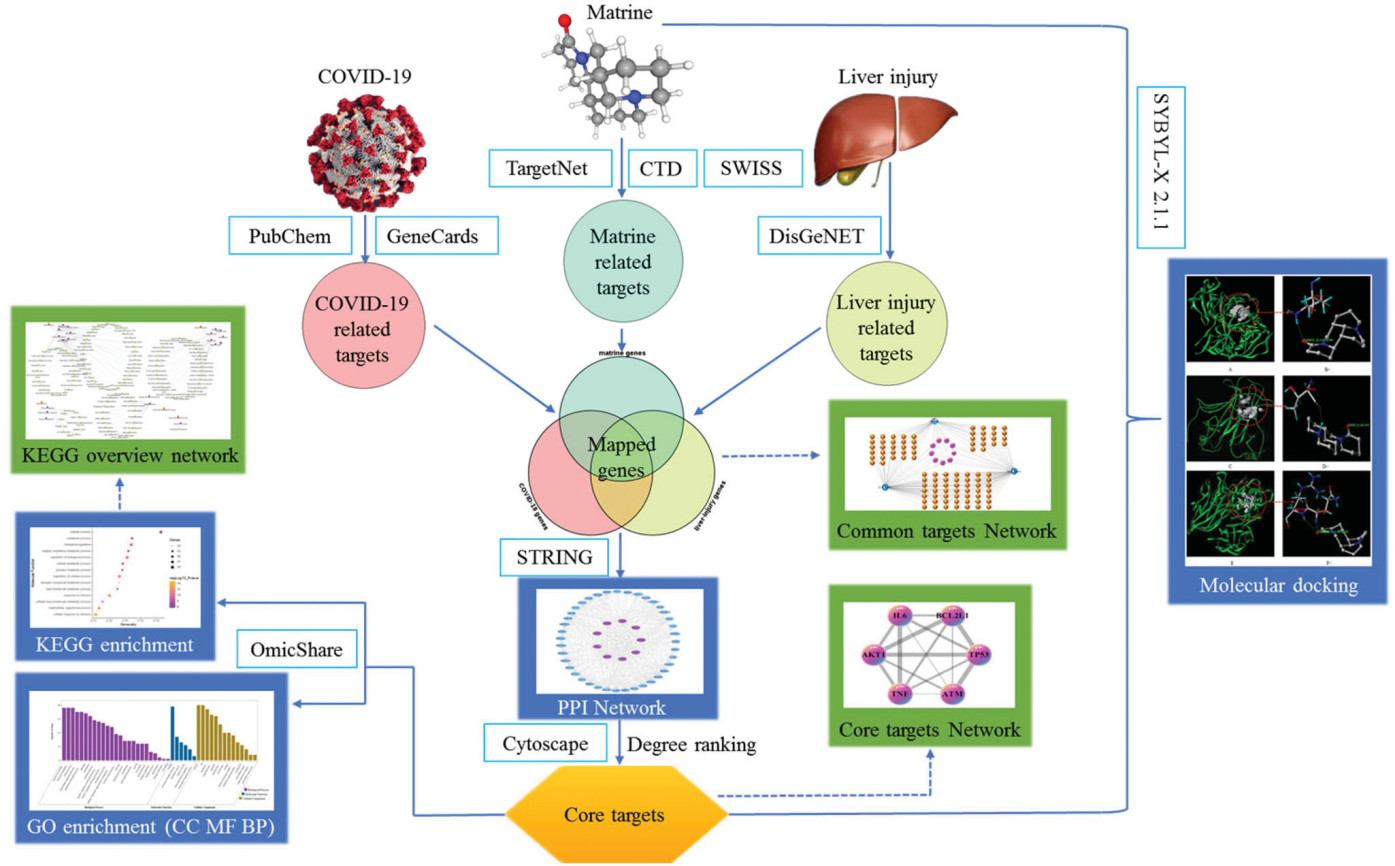
The schematic illustration of this study.

Matrine is a bioactive ingredient extracted from the traditional Chinese herb *Sophora flavescens Ait*. Figure 1 showed the chemical structure of matrine. It has a wide spectrum of pharmacological effects, including the treatment of viral hepatitis, chronic liver diseases (Tao & Wang, 1992; Long et al., 2004), antiviral, anti-inflammatory (Lin et al., 1997), immunoinhibitory (Liang et al., 2020), anti-fibrotic (Zhang et al., 2001), and anti-tumor activities (Shen et al., 2005; Ma et al., 2008) without causing significant toxicity or side effects (Zhu et al., 2003). Matrine is an ancillary drug used clinically to protect liver function and treat tumors in China (Wan et al., 2009). Remarkably, matrine played an important role in the fight against COVID-19. (Sun et al., 2020) Sun et al. in vivo animal experiments showed that matrine could reduce the pathological damage of lung tissue in COVID-19 model mice, reduce the lung index, the contents of IL-6, IL-10, TNF-α, and viral load in lung tissue, and increase the percentage of peripheral blood lymphocytes, thus enhancing the immune capacity of the body. (Yang et al., 2020) Yang et al. found that matrine can shorten the nucleic acid negative turning time of patients with COVID-19 significantly. Chest CT showed that lung lesions were well absorbed, especially for reticular and fibrotic lesions, and no obvious adverse reactions were found in all age groups during the use. Both animal and clinical experiments have confirmed that matrine has a significant therapeutic effect on COVID-19. In this study, network pharmacology and molecular docking technology were used to explore the mechanism of matrine in the treatment of COVID-19 combined with liver injury, and to provide scientific basis for matrine in the prevention and treatment of COVID-19. The schematic illustration of this study is shown in Figure 2.

## 2. Materials and methods

### 2.1. Screening of potential targets for matrine

Four databases were conducted to identify potential targets related to matrine. The four databases included The Comparative Toxicogenomics Database (http://ctdbase.org/, CTD), Swiss Target Prediction (http://www.swisstargetprediction.ch/, SWISS), TargetNet web server (http://targetnet.scbdd.com/home/index/, TargetNet) and SymMap database (https://www.symmap.org/, SymMap). CTD database is a public resource for toxicogenomic information manually curated from the peer-reviewed scientific literature, providing key information about the interactions of environmental chemicals with gene products and their effect on the human disease (Davis et al., 2017). In this study, the CTD database was used to obtain matrine related targets, which are supported by the relevant research literature. Swiss Target Prediction and the TargetNet web server are widely used in the research of Chinese medicine mechanisms and potential therapeutic targets (Yao et al., 2016; Daina et al., 2019). The prediction of the potential targets of matrine was performed by SWISS and TargetNet based on the chemical similarities and QSAR model. SymMap database provides massive descriptive information on herbs, TCM symptoms, MM symptoms, ingredients, targets, and diseases (Wu et al., 2019). We use SymMap database to supplement matrine related targets that are not obtained in the above three databases finally.

### 2.2. Screening of potential targets for COVID-19

The targets related to COVID-19 were acquired from GeneCards (https://www.genecards.org/) and PubChem (https://pubchem.ncbi.nlm.nih.gov/). These two databases illuminate the relationship between targets and disease from different perspectives. GeneCards is a comprehensive, freely available database, which provides information about targets related to disease, gene expression, gene function, protein-protein interactions, pathways, and so on (Fishilevich et al., 2016). The search conditions were set to ‘gene’ and ‘Homo sapiens,’ and the authenticity of the related genes was determined by the literature search. On the other hand, in response to the COVID-19 epidemic, PubChem has set up a special exhibition project. The project is an international, community-driven effort. It aims to establish a knowledge repository on virus-host interaction mechanisms specific to the COVID-19 virus. We can click ‘browse COVID-19 data available in PubChem’ on the PubChem search page to obtain COVID-19 related targets.

### 2.3. Screening of potential targets for liver injury

We used the online tool DisGeNET to find potential therapeutic targets for liver injury. DisGeNET is a discovery platform containing one of the largest publicly available collections of genes and variants associated with human diseases (Janet et al., 2015; Janet et al., 2017; Janet et al., 2020). By searching the keyword ‘Liver Failure,’ ‘Liver Dysfunction,’ ‘Injury of liver,’ and ‘Decreased liver function,’ then selecting the ‘Evidences for Gene-Disease Associations’ option, the targets related to liver injury were collected.

### 2.4. Screening of potential therapeutic targets of matrine for COVID-19 and liver injury

Firstly, All the targets were standardized as gene names and UniProt IDs utilizing the UniProtKB (https://www.uniprot.org/) database with the ‘Homo sapiens’ species (Apweiler et al., 2004). Then, the online Venn analysis tool (http://www.ehbio.com/ImageGP/index.php/Home/Index/VennDiagram.html) was used to analyze the related targets of matrine, COVID-19, and liver injury, so as to obtain the common target of matrine in the treatment of COVID-19 combined with liver injury.

### 2.5. Construction of matrine-COVID-19-liver injury common target network

Visual matrine-COVID-19-liver injury common targets network was established based on aforementioned data sets through Cytoscape 3.7.1 (http://www.cytoscape.org/) to reflect the complex relationships among matrine, COVID-19, liver injury and their related targets. Cytoscape 3.7.1 is an open-source software platform that is used for visualizing complicated biomolecular networks and integrating different types of attribute data (Kohl et al., 2020). In the network, nodes represent the matrine, COVID-19, liver injury, and their related targets, while the connections between the nodes represent the interactions between these biological analyses. The degree value of the molecular represents the number of connections between the molecular and target in the core architecture of the network (Tang et al., 2020).

### 2.6. Construction of PPI network and screening of core targets

In this study, Search Tool for the Retrieval of Interacting Genes (STRING, https://string-db.org/) (Szklarczyk et al., 2017) was used to collect possible protein–protein interactions (PPI) by uploading the common targets that related to matrine, COVID-19, and liver injury. Species were limited to ‘Homo sapiens’ with a confidence score >0.4. Then, Cytoscape 3.7.1 software was used to construct the PPI networks (Shannon et al., 2003). A network analyzer in Cytoscape was utilized in analyzing topological parameters of mean and maximum degrees of freedom in the PPI network (Su et al., 2019). The core targets were screened according to the value of the degrees. The larger the value is, the more likely the target is to become the core target of matrine in the treatment of COVID-19 combined with liver injury. In this study, the top six targets with the largest degree were screened as core targets.

### 2.7. Gene ontology and pathway analysis

Gene Ontology (GO) is an internationally standardized classification system of gene functions. It defines concepts related to gene function and the interrelationships among the functions of different genes (Zhang et al., 2013). In the present study, the GO describes the functions of the core targets of matrine in the treatment of COVID-19 and liver injury in terms of the molecular function, the cellular component involved, and the biological process affected (Ashburner et al., 2000). KEGG pathway analysis is a collection of databases describing biological pathways, genomes, drugs, and diseases. It can be used to study the biological effects and multidimensional pharmacological mechanism of the core targets at the pathway level. The OmicShare platform2 (http://www.omicshare.com/tools, OmicShare) was adopted to perform GO and KEGG pathway analysis and visualize the bubble chart in this study. During this procedure, the significance level was set to 0.01, and the organism was selected as Homo sapiens.

### 2.8. Matrine – core targets molecular docking

Molecular docking technology was used to verify the affinity between matrine and the crystal structure of the core target protein. The three-dimensional (3D) structure of the core targets of matrine in the treatment of COVID-19 combined with liver injury was downloaded from the RCSB PDB database (https://www.rcsb.org/) (Joosten et al., 2011). PubChem database (https://pubchem.ncbi.nlm.nih.gov/) was used to download the two-dimensional (2D) structures of matrine. The 2D structure was saved in mol2 format as docking ligands, and energy minimize was carried out to get reasonable conformations using default parameters of SYBYL-X 2.1.1. All the atoms in the target protein were protonated by setting Koll man-all atom charges. Finally, the protein structure was optimized by energy minimization by incorporating the Tripos force field (Clark et al., 1989). The docking was carried out by employing the Surflex dock module of licensed software SYBYL-X 2.1.1. The default values are set to blot (1.0) and threshold (0.5). The ligands were automatically docked into the binding sites, presenting an empirical scoring function (Malathi et al., 2016; Thillainayagam et al., 2019). The stable conformation of low energy between ligand and receptor indicates that there is a great possibility of interaction between them. Generally, binding energy ≤ −5 kJ/mol is used as the screening standard. In this Surflex dock, The best-docked ligand pose was identified by considering the Total Score (T-score), which is converted into the binding free energy (formula Δ*G*_0_ = − 2.303 *RT* × total score, where *R* is the ideal gas constant of molecule and *t* is the thermodynamic temperature of an ideal gas).

### 2.9. Matrine – core targets experimental validation

#### 2.9.1. Animal experiment

Fifty male C57BL/6 mice, 6 to 8-weeks-old, weighing 18–22 g, were purchased from Beijing Vital River Laboratory Animal Technology Co. Ltd. (Beijing, China). All experiments were approved by the Ethics Committee of the Institute of Chinese Materia Medica (2020D038). The mice were kept in the room with a suitable environment (12-h light/dark cycle, room temperature at 23 ± 3 °C and 50 ± 10% relative humidity) and supplied with normal water and food. Mice were bred for 7 days before experiments. Matrine sodium chloride injection (MSCI) was obtained from Hubei Kang qin Pharmaceutical Co., Ltd. (Hubei, China) (no. 181104).

In this study, mice were divided into 10 groups (*n* = 5): (1) normal mice for liver group (CTR-liver), (2) 10 mg/kg of LPS-sensitized liver injury mice (LPS-liver), (3) 10 mg/kg of LPS-sensitized mice treated with 36.67 ml/kg of MSCI (LPS-liver + MSCI 36.67), (4) 10 mg/kg of LPS-sensitized mice treated with 18.33 ml/kg of MSCI (LPS-liver + MSCI 18.33), (5) 10 mg/kg of LPS-sensitized mice treated with 9.17 ml/kg of MSCI (LPS-liver + MSCI 9.17), (6) normal mice for lung group (CTR-lung), (7) 50 mg/kg of LPS-sensitized lung injury mice (LPS-lung), (8) 50 mg/kg of LPS-sensitized mice treated with 36.67 ml/kg of MSCI (LPS-lung + MSCI 36.67), (9) 50 mg/kg of LPS-sensitized mice treated with 18.33 ml/kg of MSCI (LPS-lung + MSCI 18.33), and (10) 50 mg/kg of LPS-sensitized mice treated with 9.17 ml/kg of MSCI (LPS-lung + MSCI 9.17). CTR and LPS groups were intraperitoneally injected 100 μL of normal saline. While LPS + MSCI 36.67, LPS + MSCI 18.33, and LPS + MSCI 9.17 groups were intraperitoneally injected 36.67, 18.33, and 9.17 ml/kg of MSCI. We defined it as a high-dose group (H), medium-dose group (M), and low-dose group (L) of MSCI. All samples were administered for 7 days prior to LPS injection. For liver injury groups, 10 mg/kg of LPS was intraperitoneally injected. After 2 h, mice were anesthetized, and liver tissue was immediately collected. For lung injury groups, 50 mg/kg of LPS was intraperitoneally injected. After 6 h, mice were anesthetized, and lung tissue was immediately collected.

#### 2.9.2. Real-time RT-PCR

After dissection of the mice, the lung or liver tissue was collected and stored at −80 °C until further use. RNA was isolated using TRIzol Reagent (Thermo Fisher Scientific Inc.). The isolated RNA was dissolved in 20 μl of diethylpyrocarbonate (DEPC) water and stored at −80 °C until further use. Real-time RT-PCR was conducted by using One Step SYBR RT-PCR Kit (Takara Bio Ltd., China). The reaction volume was 20 μL. the reaction system contained 2 μl sample RNA, One Step SYBR GREEN 16.4 μl, 10 pmol primers, 0.8 μl each. Reaction conditions: 42 °C 5 min, 95 °C 10 s, 95 °C 5S, 60 °C 34S, a total of 40 cycles. PCR products were analyzed using QuantStudioTM Design & Analysis software v1.5.1 (QuantStudioTM 5 Real-time instrument, Thermo Fisher Scientific, USA). The upstream and downstream primers for AKT1, TP53, TNF, IL6, BCL2L1, ATM, and internal reference GAPDH are shown in Table 1. The primers were provided by Beijing AuGCT DNA-SYN Biotechnology Co., Ltd. In this study, the relative quantitative 2^−△△CT^ method was used, and GAPDH was used as an internal control. The relative concentration was expressed as ± s. Statistical analysis of the results was conducted with GraphPad Prism 7.0 software using one-way ANOVA analyses followed by Tukey’s multiple comparison procedures.

**Table 1.** List of upstream and downstream primers.

Name	Oligo	Primer sequence	Predicted size (bp)	GenBank accession
AKT1	Forward primer	5′-CTGAGACTGACACCAGGTATTTCG-3′	102	NM_009652.3
	Reverse primer	5′-GCTCACTGTCCACACACTCCAT-3′		
TP53	Forward primer	5′-CGCTGCTCCGATGGTGAT-3′	100	AB020317
	Reverse primer	5′-GGCGAAAAGTCTGCCTGTCT-3′		
TNF	Forward primer	5′-AGGACCCAGTGTGGGAAGCT-3′	100	NM_001278601
	Reverse primer	5′-AAAGAGGAGGCAACAAGGTAGAGA-3′		
IL6	Forward primer	5′-GGAGCCCACCAAGAACGATA-3′	80	NM_001314054
	Reverse primer	5′-AAGGCAACTGGATGGAAGTCTCT-3′		
BCL2L1	Forward primer	5′-GGCGGCTGGGACACTTT-3′	91	NM_001355053
	Reverse primer	5′-TCAGGAACCAGCGGTTGAAG-3′		
ATM	Forward primer	5′-TATTAATGTTCCAAGTCCTGGTCTGT-3′	122	NM_007499
	Reverse primer	5′-CAGCCCCCGAGACAAGAAT-3′		
GAPDH	Forward primer	5′-TCCTCGTCCCGTAGACAAAATG-3′	69	NM_001289726
	Reverse primer	5′-GTGACCAGGCGCCCAAT-3′		

## 3. Results

### 3.1. Potential therapeutic targets of matrine for COVID-19 and liver injury

**Figure 3.**
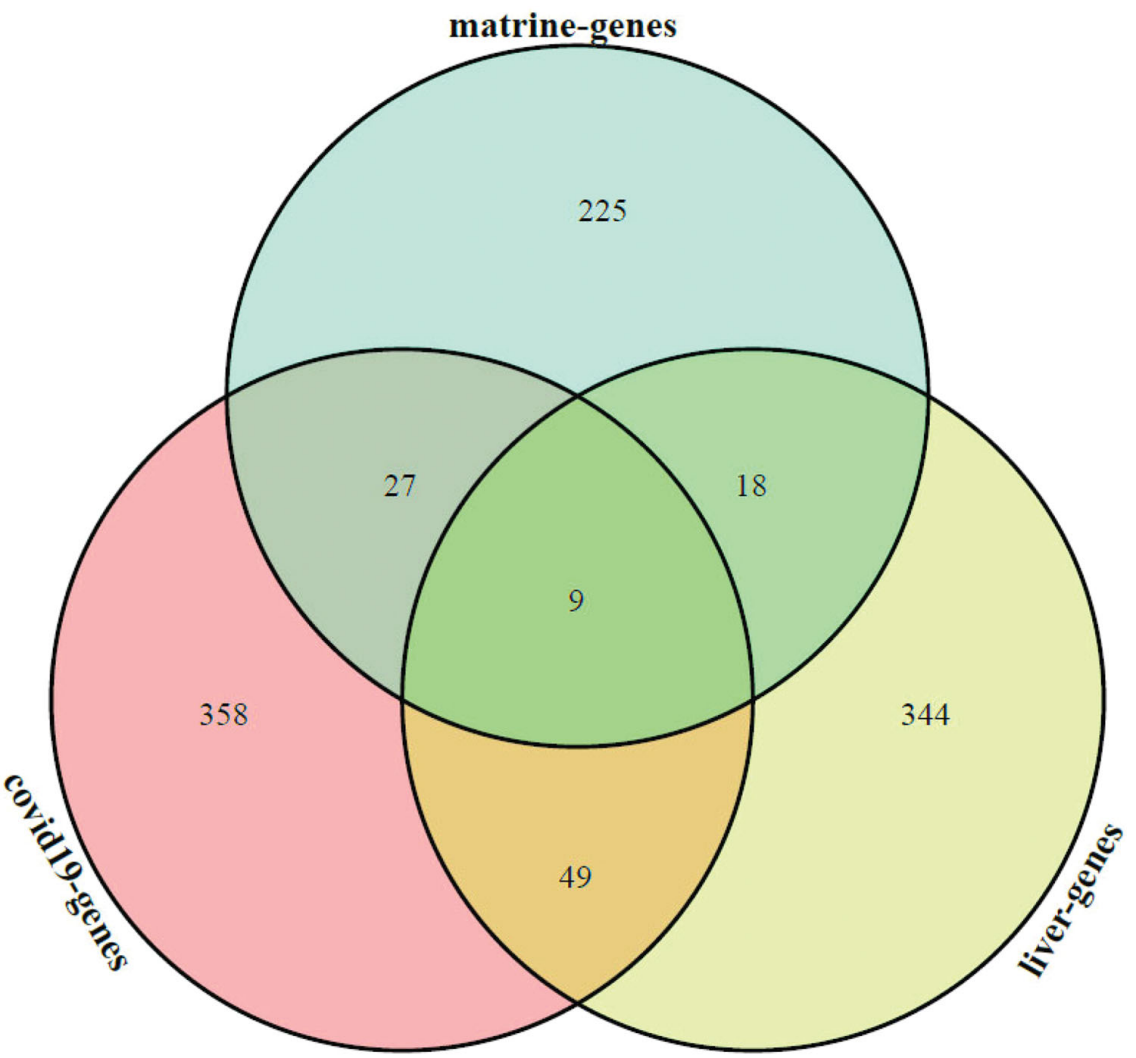
Venn analysis diagram of matrine and COVID-19 with liver injury. Green section represents the potential targets of matrine, red section represents the potential targets of COVID-19 and yellow section represents the potential targets of liver injury.

Based on the aforementioned target fishing approach, a total of 279 targets were predicted to interact with matrine, a total of 443 targets were predicted to interact with COVID-19, and a total of 420 targets were predicted to interact with liver injury. The potential targets of matrine, COVID-19, and liver injury were obtained by Venn analysis, and 9 common targets of matrine in the treatment of COVID-19 and liver injury were obtained. The Venn analysis diagram is shown in Figure 3.

### 3.2. Matrine-COVID-19-Liver injury common target network construction and analysis

**Figure 4.**
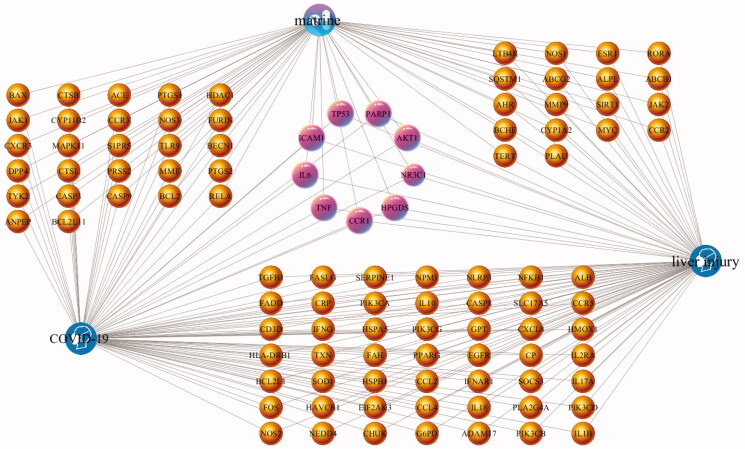
The matrine-COVID-19-liver injury common target network generated in this study. The purple capsule-shaped nodes represent matrine, the blue head-shaped nodes represent the diseases (COVID-19 and liver injury), the yellow nodes and pink nodes represent the potential targets, while the edges represent the interactions between nodes.

In order to more intuitively present the network topology status of matrine treatment of COVID-19 combined with liver injury, a matrine-COVID-19-liver injury common target network was constructed. In the network, the purple capsule-shaped nodes represent matrine. The blue head-shaped nodes represent the diseases (COVID-19 and liver injury). The pink target clusters in the center of the network are 9 common targets among matrine, COVID-19, and liver injury, which are AKT1, CCR1, HPGDS, ICAM1, IL6, NR3C1, PARP1, TNF, and TP53. Then the yellow target clusters between matrine node and COVID-19 node are 22 common targets between matrine and COVID-19, the yellow target clusters between matrine node and liver injury node are 18 common targets between matrine and liver injury, the yellow target clusters between COVID-19 node and liver injury node are 49 common targets between COVID-19 and liver injury. The matrine-COVID-19-liver injury common target network is shown in Figure 4.

### 3.3. PPI network construction and core targets

**Figure 5.**
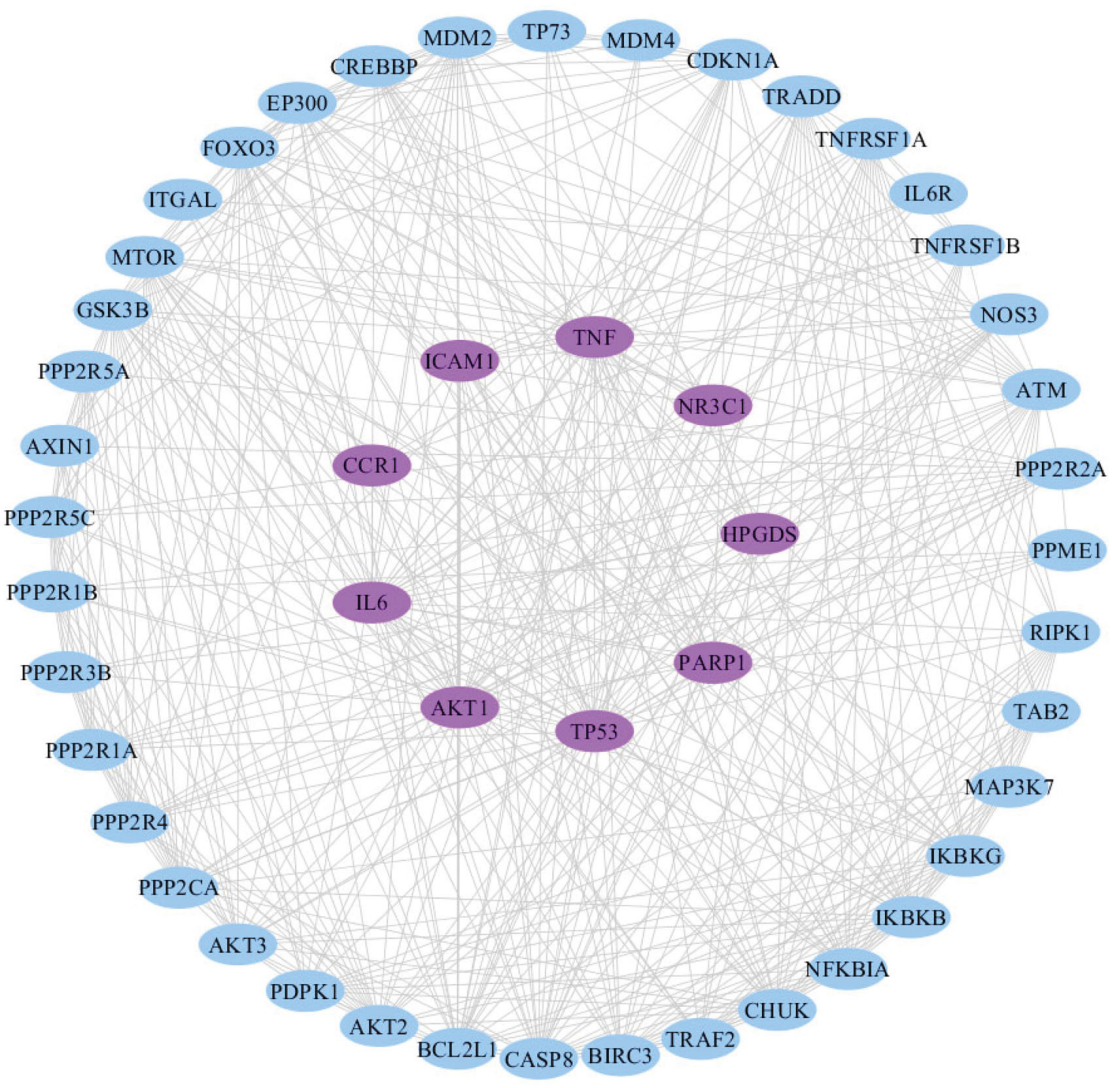
The PPI network generated in this study. The pink nodes represent the 9 common targets, the blue nodes represent the related active proteins obtained by the STRING Tool, while the edges represent the interactions between nodes.

Proteins tend to form macromolecular complexes through interactions to complete the biological functions within cells, but seldom achieve assigned functions solely (Shekarappa et al., 2019). Hence, the study of protein-protein interaction and its interaction network (PPI) is very important for understanding cell tissue, biological process, and function. In this study, a PPI network model graph was constructed with the 9 common targets among matrine, COVID-19, and liver injury. As shown in Figure 5, the PPI network finally contains 49 nodes and 472 edges. The pink nodes represent the 9 common targets, the blue nodes represent the related active proteins obtained by the STRING Tool, the edges represent the interaction between the active proteins and proteins. Results of the network topology analysis are as follows: network density (0.401), network heterogeneity (0.416), and shortest paths (2352, 100%). The average degree of nodes is 19.26531, and there are 22 nodes larger than the average degree. The average betweenness centrality of nodes is 0.01369, and there are 15 nodes larger than the average betweenness centrality.

**Figure 6.**
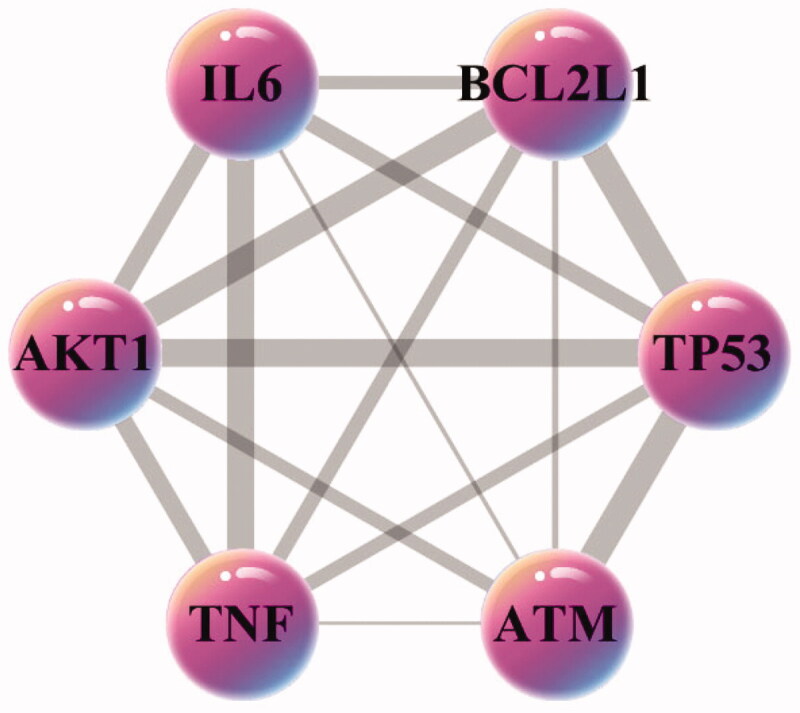
The core targets network generated in this study. The pink nodes represent the core targets, the edges represent the interactions between nodes.

The key core nodes are screened based on the topological properties of the degree of network nodes. The results showed that the six targets of AKT1, TP53, TNF, IL6, BCL2L1, and ATM were in the front of degree ranking (degree > 27), and they were the pivotal nodes in the network, indicating that they might be the core targets of the pharmacological mechanism of matrine in treatment of COVID-19 combined with liver injury. In this study, the combined scores of 6 core targets were obtained from a string database, and then the core targets and combined scores were imported into Cytoscape software to construct the core target network. In the network, the pink nodes represent the core targets, the edges represent the interactions between nodes, while the edge thickness varies according to the combined score. The core target network is shown in Figure 6.

### 3.4. Analysis of GO enrichment

**Figure 7.**
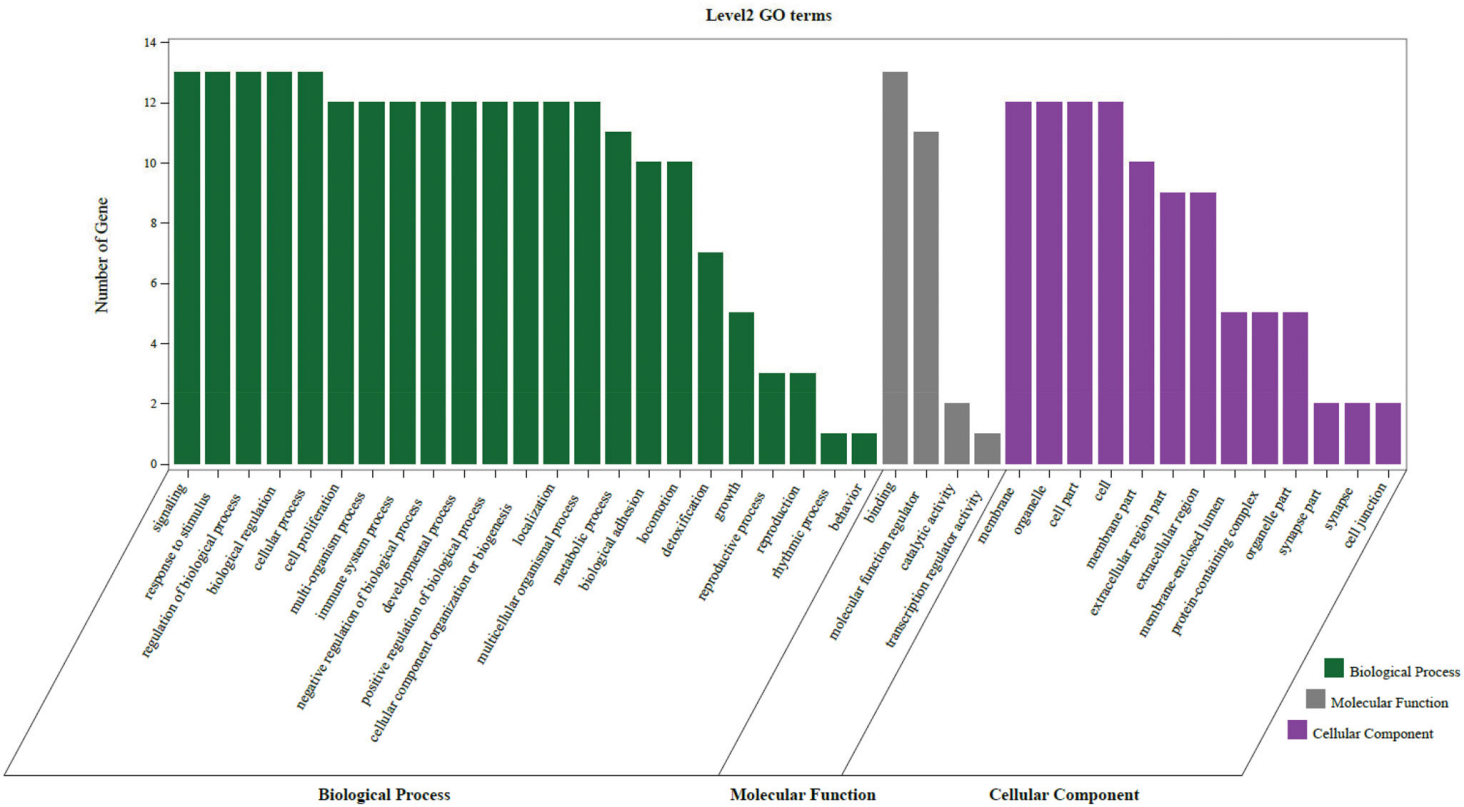
The second level GO functional enrichment analysis statistics of core targets. The vertical axis represents the number of gene, the horizontal axis represents the level 2 GO terms, the green bars represent biological process, the gray bars represent molecular function, the purple bars represent cellular component.

**Figure 8.**
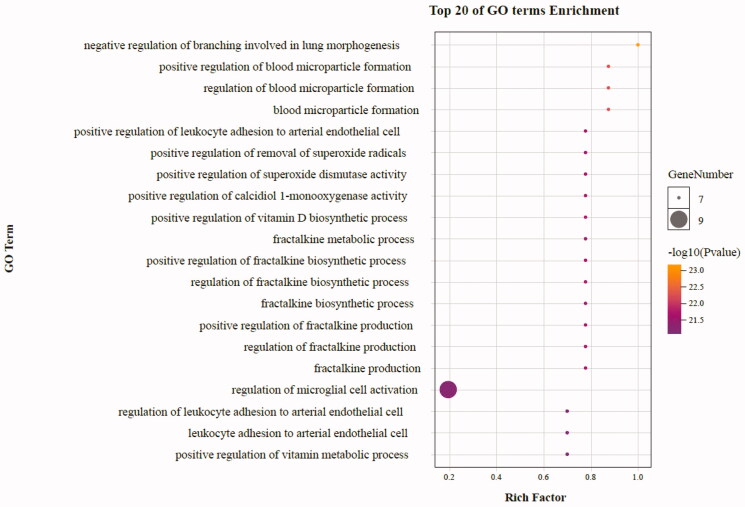
The top 20 GO enrichments in BP. The vertical axis represents the GO term name, the horizontal axis represents the rich factor, the size of the dot indicates the number of genes expressed in the GO term, and the color of the dot corresponds to the different *p*-value range.

**Figure 9.**
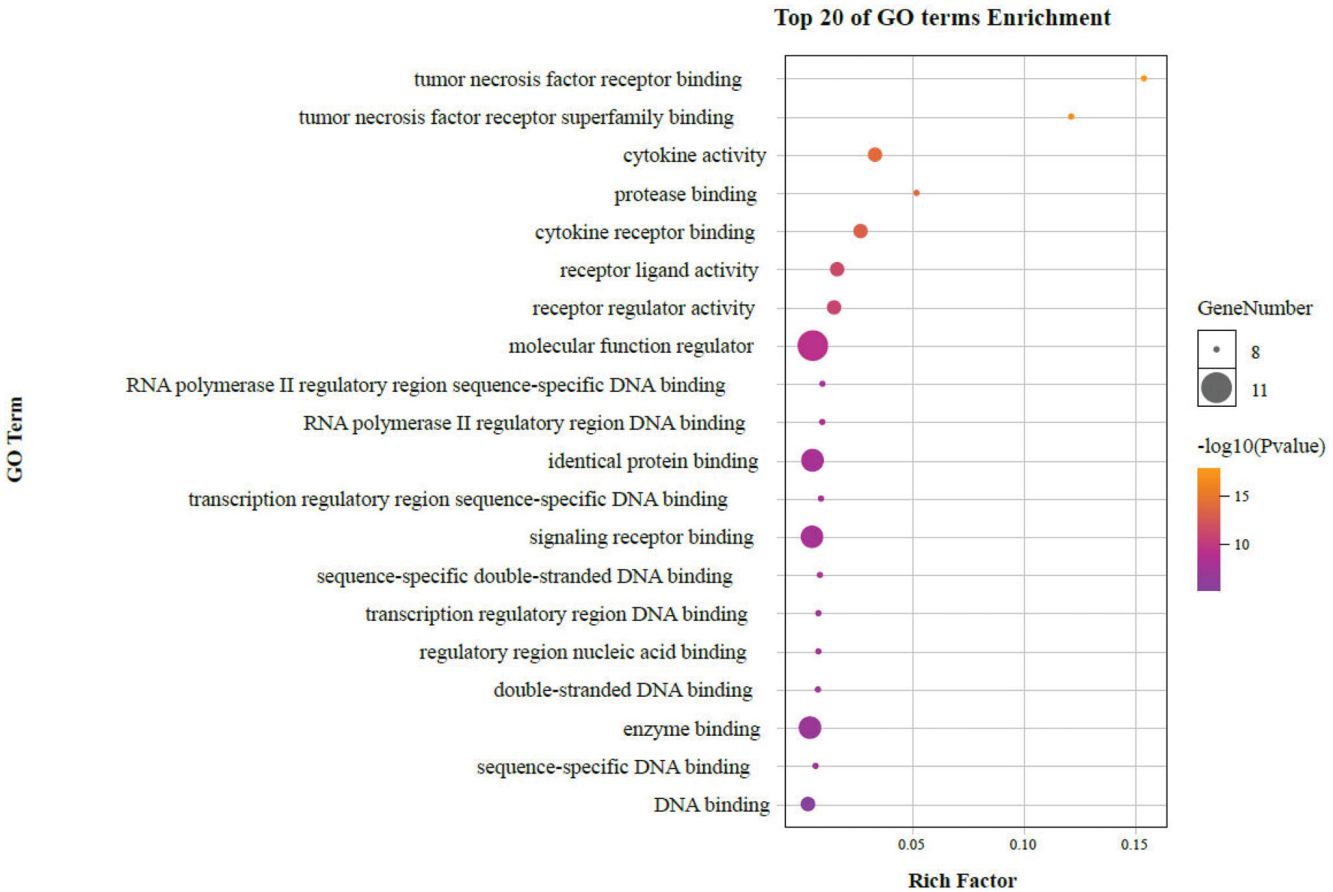
The top 20 GO enrichments in MF. The vertical axis represents the GO term name, the horizontal axis represents the rich factor, the size of the dot indicates the number of genes expressed in the GO term, and the color of the dot corresponds to the different *p*-value range.

**Figure 10.**
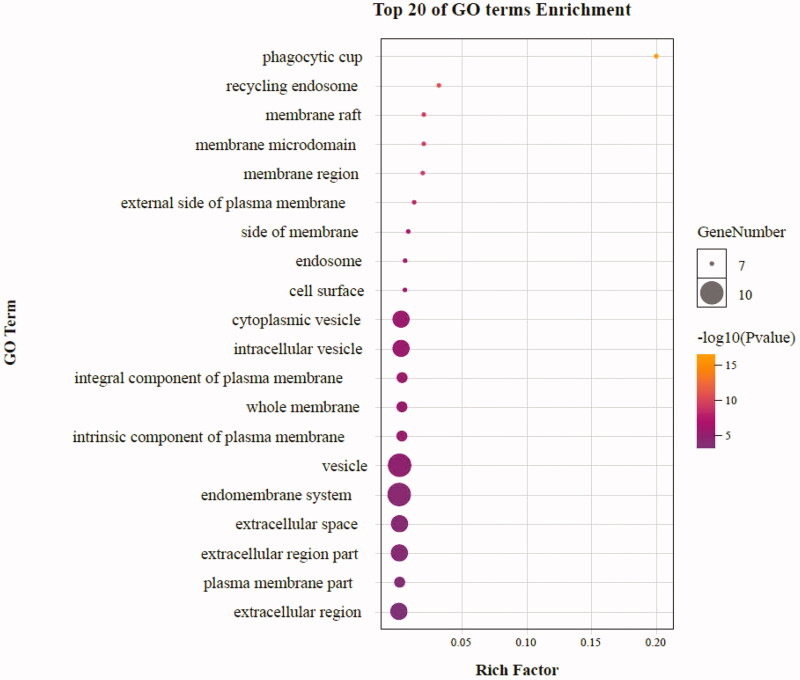
The top 20 GO enrichments in CC. The vertical axis represents the GO term name, the horizontal axis represents the rich factor, the size of the dot indicates the number of genes expressed in the GO term, and the color of the dot corresponds to the different *p*-value range.

As highlighted in Figure 7, gene ontology enrichment analysis consisted of three parts, BP (biological process), MF (molecular function), and CC (cellular component). Different categories of biological process, molecular function, and cellular component were represented by a green, gray and purple bar, respectively. The height of the bar represented the number of genes observed in the category. Each *p*-value of enrichment results was calculated (*p*-value < .01 was considered to be significantly enriched), ranking *p*-values according to the order from small to large. The top 20 BP, MF, CC terms are displayed in bubble charts as shown in Figures 8–10, respectively. The enrichment results showed that there were 2281 enrichment processes are related to the biological processes which cover negative regulation of branching involved in lung morphogenesis, positive regulation of blood microparticle formation, regulation of blood microparticle formation, blood microparticle formation, positive regulation of leukocyte adhesion to arterial endothelial cell; 122 enrichment results are related to molecular function, which includes tumor necrosis factor receptor binding, tumor necrosis factor receptor superfamily binding, cytokine activity, protease binding, cytokine receptor binding; 135 enrichment results in the related items of cell composition, involving phagocytic cup, recycling endosome, membrane raft, membrane microdomain, membrane region, etc. These results help to elucidate the biological function changes in the body after administration of matrine in the treatment of COVID-19 combined with liver injury.

### 3.5. Analysis of KEGG pathway enrichment

**Figure 11.**
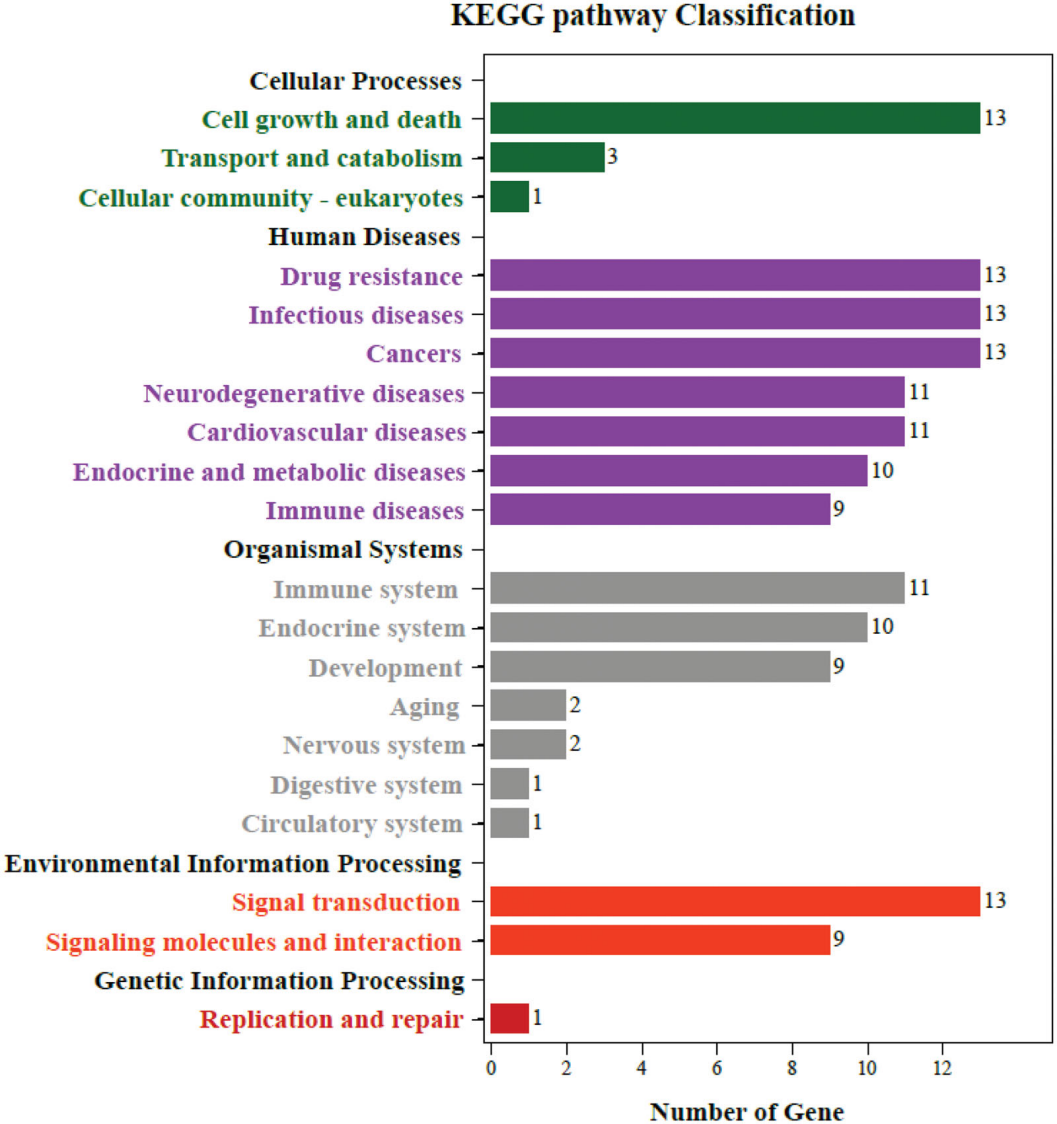
The classification bar chart of KEGG results. The vertical axis represents the name of pathway classification, the horizontal axis represents the number of gene.

**Figure 12.**
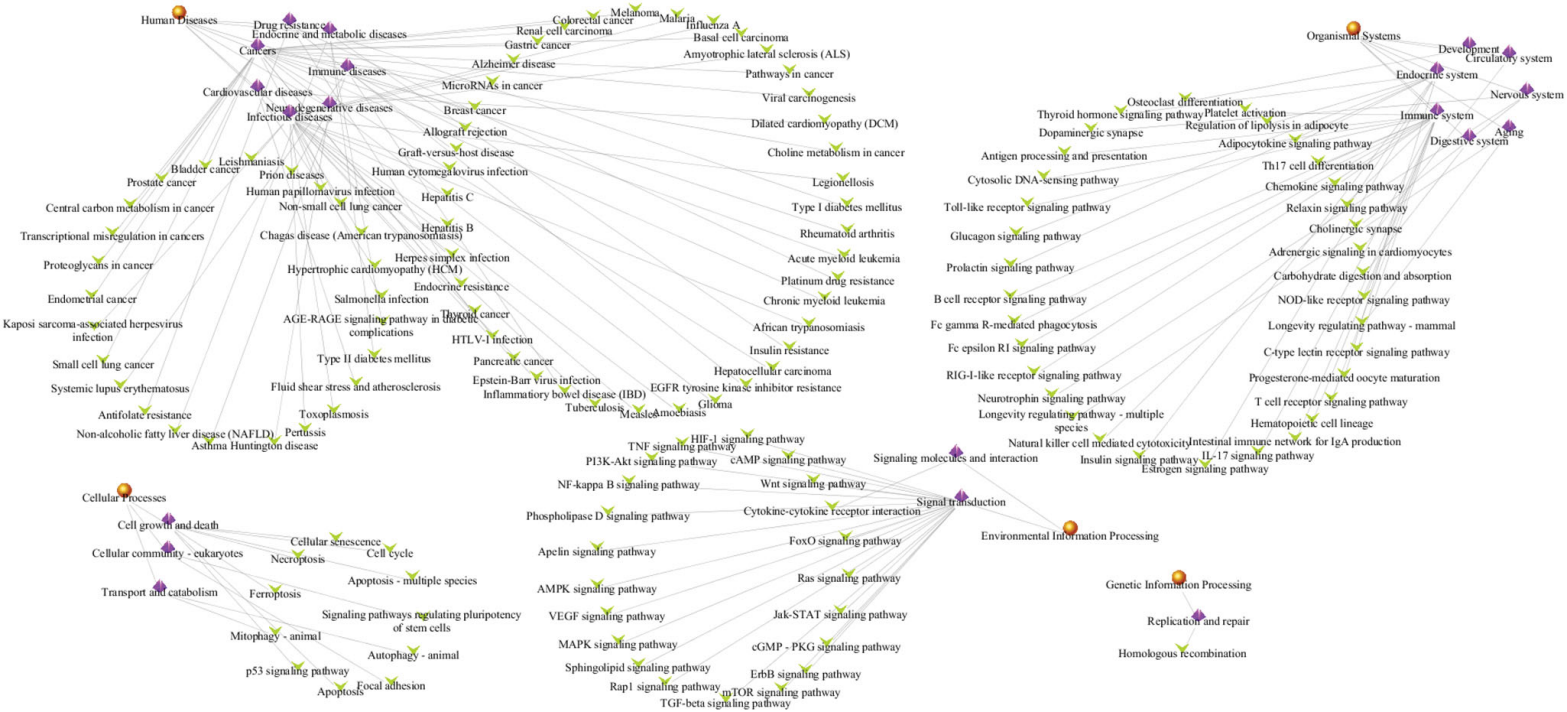
The overview map of functionally grouped network of KEGG results enriched categories. The first level classification of KEGG results are shown with yellow circular nodes; purple diamond nodes represent the second level classification of KEGG results and green V nodes are KEGG pathways.

**Figure 13.**
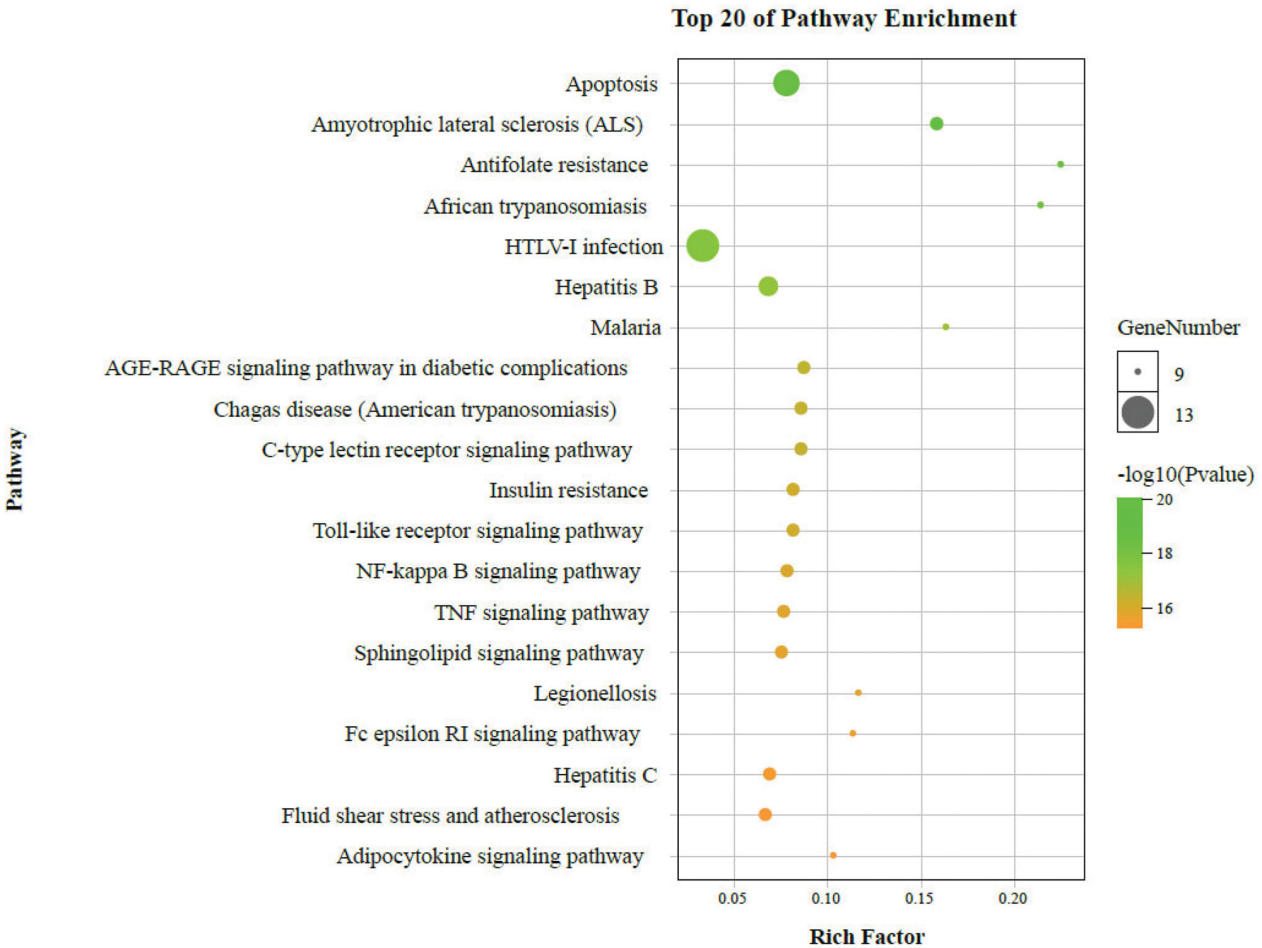
Pathway enrichment point diagram of 36 target genes. The vertical axis represents the pathway name, the horizontal axis represents the rich factor, the size of the dot indicates the number of genes expressed in the pathway, and the color of the dot corresponds to the different *p*-value range.

To further reveal the potential therapeutic mechanism of matrine on COVID-19 combined with liver injury, we conducted KEGG pathway enrichment analysis on 6 core targets and screened 133 pathways that were significantly correlated with the target genes (*p* < .01). The classification bar chart of KEGG results was displayed in Figure 11. The overview map of KEGG results was displayed in Figure 12. The top 20 pathway with lower *p*-values and more genes enrichment are listed in Table 2, including HTLV-I infection, Apoptosis, Platinum drug resistance, Hepatitis B, Transcriptional misregulation in cancers, Amyotrophic lateral sclerosis (ALS), p53 signaling pathway, Pancreatic cancer, Chronic myeloid leukemia, EGFR tyrosine kinase inhibitor resistance, Human cytomegalovirus infection, Cellular senescence, Small cell lung cancer, AGE-RAGE signaling pathway in diabetic complications, C-type lectin receptor signaling pathway, Chagas disease (American trypanosomiasis), PI3K-Akt signaling pathway, Toll-like receptor signaling pathway, Insulin resistance, and NF-kappa B signaling pathway. These pathways mainly involve Infectious diseases, Drug resistance, Cancers, Neurodegenerative diseases, Endocrine and metabolic diseases, Cell growth and death, Signal transduction, and Immune system. The senior bubble map visually showed these signifcantly enriched pathways (Figure 13). The size and color of the nodes in the bubble graph were decided by the number of associated genes and the *p*-values. The size of the nodes indicated how many target genes are associated, and the colors from purple to yellow reflected the *p*-values from high to low.

**Table 2.** Classification and information on core targets-related KEGG pathways, direct (Top 20).

Level 1 classification	Level 2 classification	Pathway	*p*-Value	Genes count
Human diseases	Infectious diseases	HTLV-I infection	8.37E-09	6
		Hepatitis B	1.75E-06	4
		Human cytomegalovirus infection	2.35E-05	4
		Chagas disease (American trypanosomiasis)	4.78E-05	3
	Drug resistance	Platinum drug resistance	8.68E-08	4
		EGFR tyrosine kinase inhibitor resistance	1.69E-05	3
	Cancers	Transcriptional misregulation in cancers	3.86E-06	4
		Pancreatic cancer	1.40E-05	3
		Chronic myeloid leukemia	1.51E-05	3
		Small cell lung cancer	3.07E-05	3
	Neurodegenerative diseases	Amyotrophic lateral sclerosis (ALS)	7.60E-06	3
	Endocrine and metabolic diseases	AGE-RAGE signaling pathway in diabetic complications	4.54E-05	3
		Insulin resistance	5.56E-05	3
Cellular processes	Cell growth and death	Apoptosis	1.04E-08	5
		p53 signaling pathway	1.19E-05	3
		Cellular senescence	3.05E-05	4
Environmental information processing	Signal transduction	PI3K-Akt signaling pathway	5.12E-05	4
		NF-kappa B signaling pathway	6.27E-05	3
Organismal systems	Immune system	C-type lectin receptor signaling pathway	4.78E-05	3
		Toll-like receptor signaling pathway	5.56E-05	3

### 3.6. Results of molecular docking

In this paper, matrine with corresponding 6 core targets were simulated by molecular docking, and the docking results were analyzed. Using SYBYL-X 2.1.1 software, matrine was observed to enter the active pocket of the six proteins, respectively (Figure 14). As shown in the figure, matrine small molecule forms hydrogen bonds with LYS268 residues on AKT1, with THR231 residues on TP53, with ARG103 and GLN102 residues on TNF, with ARG182 and ARG179 residues on IL-6, with ARG146 and ASN143 residues on BCL2L1, with LEU2046 residues on ATM. The results of molecular docking analysis showed that the binding free energy (Δ*G* in kcal/mol) value of matrine to the core target was negative, indicating that the ligand molecule could spontaneously bind to the receptor protein. Furthermore, the binding energy in this result was less than − 5 kJ/mol, which further proved the strong binding ability. The binding energies of the matrine with various core targets are shown in Table 3.

Figure 14.Molecular docking models of matrine binding to the six core targets. (A) Matrine was docked to the surface of AKT1. (B) The binding interaction between matrine and AKT1 protein. (C) Matrine was docked to the surface of TP53. (D) The binding interaction between matrine and TP53 protein. (E) Matrine was docked to the surface of TNF. (F) The binding interaction between matrine and TNF protein. (G) Matrine was docked to the surface of IL6. (H) The binding interaction between matrine and IL6 protein. (I) Matrine was docked to the surface of BCL2L1. (J) The binding interaction between matrine and BCL2L1 protein. (K) Matrine was docked to the surface of ATM. (L) The binding interaction between matrine and ATM protein.

**Figure:**
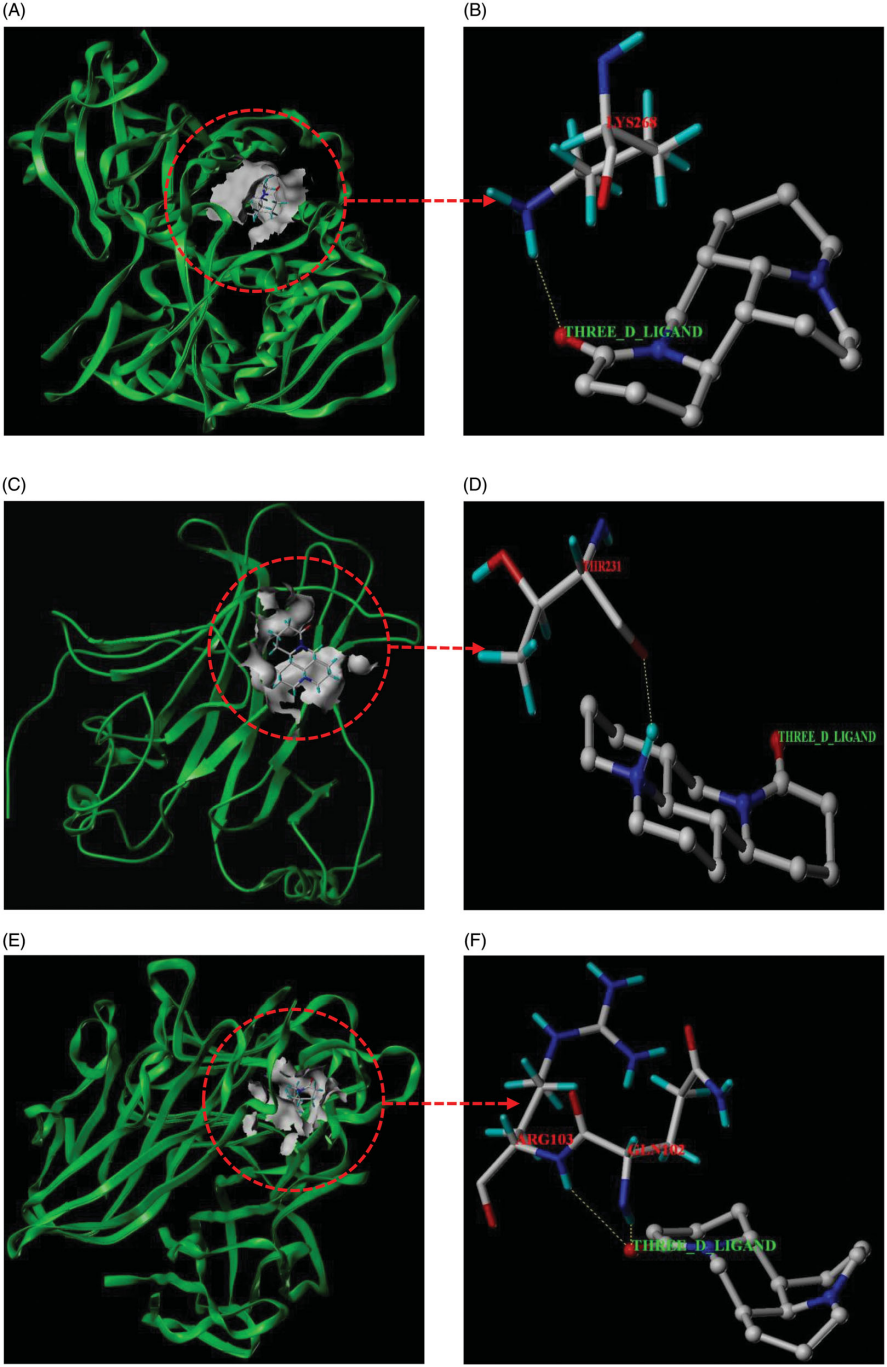

**Figure:**
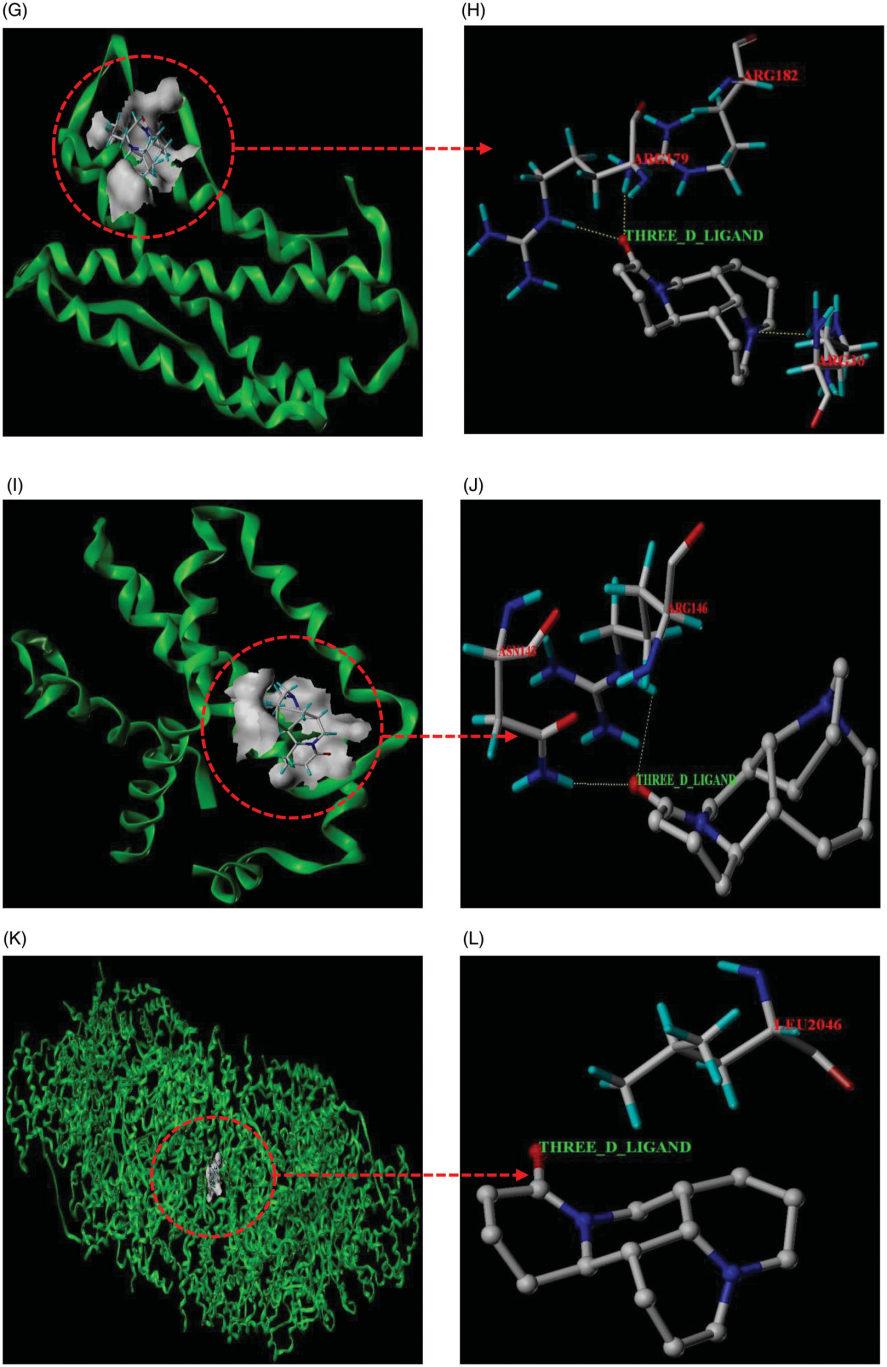

**Table 3.** The binding energy of the matrine with various core targets.

Compount	Core target	PDB ID	T-score	Bingding energy (kJ/mol)
Matrine	AKT1	6HHF	4.4657	−25.49343263
Matrine	TP53	3DCY	3.2791	−18.71946502
Matrine	TNF	3ALQ	4.1022	−23.41831277
Matrine	IL6	1ALU	3.7861	−21.61378626
Matrine	BCL2L1	3ZK6	3.4186	−19.51583152
Matrine	ATM	5NP1	4.5313	−25.86792469

### 3.7. Results of experimental verification

**Figure 15.**
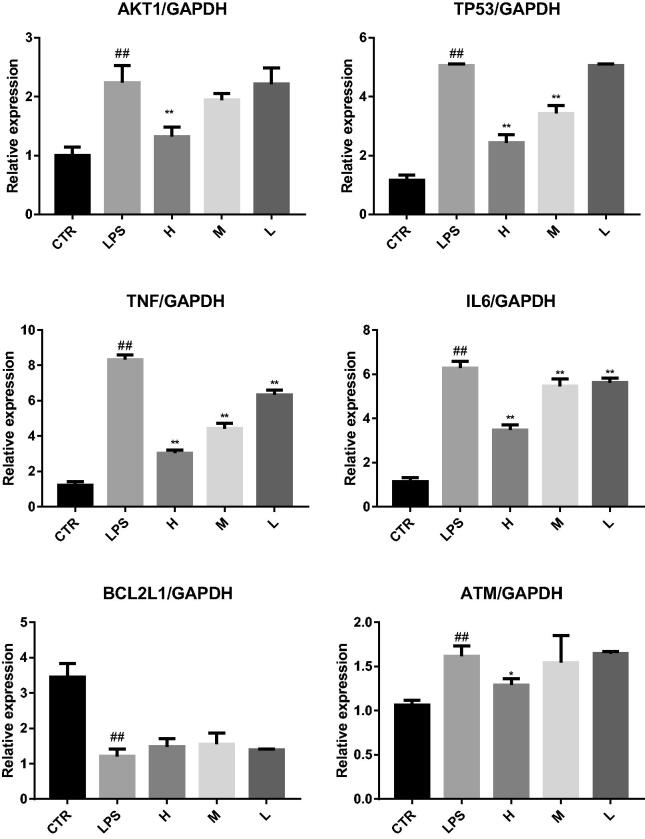
Real time fluorescent quantitative RT-PCR was used to detect the effects of MSCI on the expression of AKT1, TP53, TNF, IL6, BCL2L1, and ATM in liver tissue of mice with liver injury (##*p* < .01 vs. the CRT group; **p < .01 vs. the LPS group; *p < .05 vs. the LPS group).

**Figure 16.**
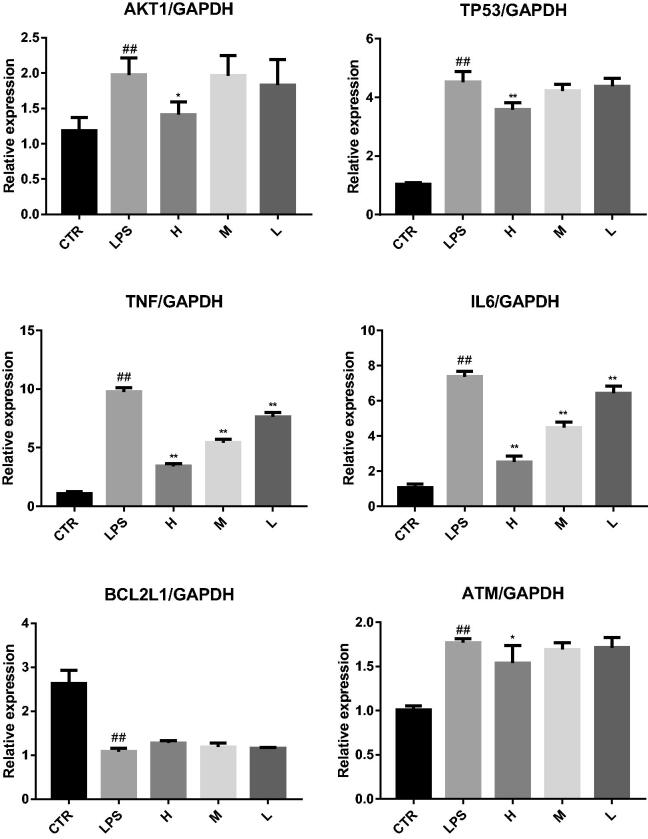
Real time fluorescent quantitative RT-PCR was used to detect the effects of MSCI on the expression of AKT1, TP53, TNF, IL6, BCL2L1, and ATM in lung tissue of mice with lung injury (##*p* < .01 vs. the CRT group; **p < .01 vs. the LPS group; *p < .05 vs. the LPS group).

Real-time fluorescent quantitative RT-PCR showed that (1) For liver injury mice, compared with the CTR group, the expression levels of AKT1, TP53, TNF, IL6, and ATM in the LPS group were significantly increased, and the expression levels of BEL2L1 were significantly decreased. After MSCI pretreatment, compared with those in the LPS group, the expression levels of AKT1, TP53, TNF, IL6, and ATM in the high-dose MSCI group (H) were significantly decreased (*p* < .05), the expression levels of TP53, TNF, IL6 and ATM in medium-dose MSCI group (M) were significantly decreased (*p* < .05), and the expression levels of TNF and IL6 in MSCI low-dose (L) group were significantly decreased (*p* < .05), but the expression levels of BEL2L1 in MSCI high, medium and low-dose groups were not significantly increased (*p* > .05). This suggests that MSCI pretreatment can reduce the expression of AKT1, TP53, TNF, IL6, and ATM in LPS induced liver tissue, as shown in Figure 15. (2) For lung injury mice, compared with the CTR group, the expression levels of AKT1, TP53, TNF, IL6, and ATM in the LPS group were significantly increased, and the expression levels of BEL2L1 were significantly decreased. After MSCI pretreatment, compared with those in LPS group, the expression levels of AKT1, TP53, TNF, IL6, and ATM in the high-dose MSCI group (H) were significantly decreased (*p* < .05), the expression levels of TNF and IL6 in medium-dose MSCI group (M) were significantly decreased (*p* < .05), and the expression levels of TNF and IL6 in MSCI low-dose group were significantly decreased (*p* < .05), but the expression levels of BEL2L1 in MSCI high, medium and low-dose groups (L) were not significantly increased (*p* > .05). This suggests that MSCI pretreatment can reduce the expression of AKT1, TP53, TNF, IL6, and ATM in LPS induced lung tissue, as shown in Figure 16.

## 4. Discussion

Although China’s domestic epidemic has been gradually controlled, the number of confirmed cases of novel coronavirus pneumonia in foreign countries is still increasing rapidly. According to Coronavirus disease (COVID-19) weekly epidemiological and operational updates report received by WHO from national authorities by 10:00 on August 23, 2020, a cumulative total of over 23 million cases and 800 000 deaths have been reported worldwide (World Health Organization, 2020). This indicates that the novel coronavirus pneumonia is still in the outbreak stage. However, with the continuous development of the epidemic, the number of clinical cases has increased dramatically. Besides causing respiratory symptoms, COVID-19 combined with liver injury has attracted extensive attention. In view of this situation, ‘The protocol for prevention, diagnosis and treatment of liver injury in coronavirus disease 2019’ was formulated by the China Digestion Association of Chinese Medical Doctor Association and Chinese Society of Hepatology of Chinese Medical Association (China Digestion Association of Chinese Medical Doctor Association, 2020). What’s more, for severe patients with COVID-19 and previous severe liver disease, especially elderly patients with comorbidities, more targeted treatment is needed. Therefore, it is of great significance to develop drugs for comorbidities.

From the point of view of a similar virus, COVID-19 combined with liver injury can not be ignored. COVID-19 virus shares 82% genome sequence similarity to SARS-CoV and 50% genome sequence homology to Middle East respiratory syndrome coronavirus (MERS-CoV). Liver impairment has been reported in up to 60% of patients with SARS (Chau et al., 2004) and has also been reported in patients infected with MERS-CoV (Alsaad et al., 2018). On the other hand, according to relevant literature reports, liver injury caused by COVID-19 may be directly caused by SARS-CoV-2 infection of hepatocytes or drug-induced (Xu, Wu, et al., 2020). Li et al. demonstrated that 50.7% had abnormal liver functions at admission in 148 patients with COVID-19, and the abnormal liver enzyme was significantly higher in cases that received ritonavir or lopinavir compared with patients with normal liver function test (Li & Fan, 2020). Chai et al. reported that ACE2, as a receptor of SARS-CoV-2, had low expression (2.6%) in hepatocytes but the high expression in cholangiocytes, suggesting that COVID-19 may damage bile duct cells, and speculate that liver injury in patients with COVID-19 may be caused by cholangiocyte dysfunction (Chai et al., 2020).

Matrine, as a clinical drug for liver function protection approved by China, has played an important role in this COVID-19 epidemic. Recent animal experiments confirmed that matrine can significantly reduce the pathological damage of lung tissue in model mice, and the inhibition rate of lung index can reach 86.86% (Sun et al., 2020). The clinical study of matrine sodium chloride injection for COVID-19 patients showed that the effective rate of 40 cases was 100% (Yang et al., 2020). The results of this study showed that there were 9 intersection targets among matrine, COVID-19, and liver injury, but proteins in the human body did not exist independently, and there was the interaction between proteins. To identify functional connections between the predicted target proteins, we construct a PPI network to predict protein-protein interactions and identified six core targets according to the degree value, namely AKT1, TP53, TNF, IL6, BCL2L1, and ATM.

In this study, GO enrichment analysis and KEGG pathways enrichment analysis were carried out in order to explore the multi-dimensional pharmacological mechanism of matrine on COVID-19 combined with liver injury. We analyzed the pathway with more annotation genes and a lower *p*-value: among these pathways, HTLV-I infection, Hepatitis B, and Human cytomegalovirus infection are mainly associated with Viral infectious diseases. According to this, we infer that matrine may have a broad-spectrum antiviral effect; While Transcriptional misregulation in cancers, Pancreatic cancer, Chronic myeloid leukemia, Small cell lung cancer, Platinum drug resistance, and EGFR tyrosine kinase inhibitor resistance is mainly related to the drug resistance mechanism of cancer and anticancer targeted drugs. These pathways are not closely related to the study of matrine against COVID-19 combined with liver injury; the C-type lectin receptor signaling pathway and Toll-like receptor signaling pathway are related to the Immune system, which are the two pathways that we pay more attention to. Because relevant studies have shown that sars-cov-2 infection can activate human immune cells, resulting in excessive aggregation of immune cells, the release of pro-inflammatory cytokines, and then lead to lung, liver, and other organ damage (Xu, Shi, et al., 2020). Therefore, it is speculated that ‘cytokine storm’ is one of the important causes of liver injury in patients with COVID-19; Furthermore, PI3K-Akt signaling pathway, NF-kappa B signaling pathway, and p53 signaling pathway are also worthy of attention. PI3K-Akt mediated NF-kappa B is a classic inflammatory signal transduction pathway, which plays an important role in regulating the level of inflammatory factors in vivo. Once PI3K-Akt signaling pathway is activated, NF-kappa B can produce a large number of inflammatory factors and aggravate the inflammatory reaction. These pathways may inhibit the excessive release of related inflammatory cytokines, so as to avoid exacerbation of patients with COVID-19 combined with liver injury due to cytokine storm. Protein 53 (p53) is an important transcription factor that is involved in the regulation of mitochondrial apoptosis. Evidence also shows that p53 protein accumulates in patients with various inflammatory liver diseases (Dibra et al., 2016; Akyol et al., 1999). p53 signaling pathway is mainly associated with cell growth and death. Inhibition of p53 signaling pathway can improve ethanol-induced hepatocyte injury by regulating the mitochondrial apoptosis pathway, and thus become an effective therapeutic strategy for treating liver injury (Yuan et al., 2020). Meanwhile, evidence showed that p53 signaling pathway plays an important role in inducing apoptosis and antivirus by regulating the synthesis of cell cycle-related proteins (Peng et al., 2020). Then, molecular docking technology was used to verify the binding of six core targets with matrine. The results showed that the binding free energy of matrine with the core target was less than −5 kJ/mol, which indicated that the ligand molecule could spontaneously bind to the receptor protein and had a strong binding force. Finally, to further confirm the results of network pharmacological analysis, real-time RT-PCR was used to detect the effects of matrine on AKT1, TP53, TNF, IL6, BCL2L1, and ATM gene expression in liver tissue of liver injury mice and lung tissue of lung injury mice. The validation experiments demonstrated that MSCI could significantly reduce the expression of AKT1, TP53, TNF, IL6, and ATM in mice with liver injury or lung injury (*p* < .05), and increase the expression of BCL2L1 to a certain extent (*p* > .05), which confirmed the rationality of network pharmacology analysis results together with the molecular docking experiment.

In this study, we investigated the underlying mechanism of matrine in treating COVID-19 combined with liver injury utilizing the approaches of integrating network pharmacology and molecular docking. Matrine can inhibit cytokine storm, maintain liver function homeostasis, regulate immunity, antivirus, and other functions through direct target action and signal pathway regulation, which embodies the characteristics of overall regulation, network regulation, and interaction. Furthermore, as an important adjuvant therapy, traditional Chinese medicine components, such as matrine, combined with modern medicine approaches would benefit patients with COVID-19, especially patients with liver injury complications, and help to overcome the current COVID-19 pandemic.
